# The *salmonella* effector Hcp modulates infection response, and affects *salmonella* adhesion and egg contamination incidences in ducks

**DOI:** 10.3389/fcimb.2022.948237

**Published:** 2022-10-03

**Authors:** Lina Song, Jia Wu, Kaiqi Weng, Fenghua Yao, Wanwipa Vongsangnak, Guoqiang Zhu, Guohong Chen, Yu Zhang, Qi Xu

**Affiliations:** ^1^ Joint International Research Laboratory of Agriculture & Agri-Product Safety of Ministry of Education, Yangzhou University, Yangzhou, China; ^2^ College of Animal Science and Technology, Yangzhou University, Yangzhou, China; ^3^ Department of Zoology, Faculty of Science, Kasetsart University, Bangkok, Thailand

**Keywords:** *Hcp*, *Salmonella* enteritidis, invasion, ovarian granulosa cells, egg contamination

## Abstract

*Salmonella* Entertidis (SE) often causes persistent infections and egg contamination in laying ducks. Hcp, the core structural and effector proteins of the Type VI Secretion System (T6SS) in SE, contributes to bacterial invasion, adhesion and virulence. However, little is known about the effect of Hcp on the host’s infection responses and egg contamination incidences in duck. Herein, we generated an *hcp* deletion mutant SE MY1△*hcp* and detected its ability to invade duck granulosa cells (dGCs) and contaminate eggs. In comparison with MY1*-*infected group, the SE adhesion decreased by 15.96% in MY1△*hcp-*infected dGCs, and the apoptosis in MY1△*hcp-*infected dGCs decreased by 26.58% and 30.99% at 3 and 6 hours postinfection, respectively. However, the expression levels of immunogenic genes *TLR4*, *NOD1*, *TNFα*, *IL-1β* and proinflammatory cytokines IL-6, IL-1β, TNF-α release were markedly lower in the dGCs inoculated with MY1△*hcp* than that of the wild type. Besides, the laying ducks were challenged with MY1 or MY1△*hcp in vivo*, respectively. The lower egg production and higher egg contamination were observed in MY1*-*infected ducks in comparison with MY1△*hcp*-infected birds. Furthermore, the host’s infection response of differentially abundant proteins (DAPs) to *Salmonella* effector Hcp was identified using quantitative proteomics. A total of 164 DAPs were identified between the MY1- and MY1△*hcp*-infected cells, which were mainly engaged in the immune, hormone synthesis, cell proliferation and cell apoptotic process. Among them, STAT3, AKT1, MAPK9, MAPK14, and CREBBP were the center of the regulatory network, which might serve as key host response regulators to bacterial Hcp. In conclusion, we demonstrated that effector Hcp contributed to not only SE invasion, induction of dGCs apoptosis, and trigger of immune responses, but also enhanced contamination incidences. Also, the STAT3, AKT1, MAPK9, MAPK14, and CREBBP were identified as host’s infection response regulators of bacterial Hcp in duck. Overall, these results not only offered a novel evidence of SE ovarian transmission but also identified some promising candidate regulators during SE infection.

## Introduction

*Salmonella* Enteritidis (SE) is among the common microbes that plague avian and human health and pose a great risk of foodborne infections, owing to their contamination of eggs and poultry. Globally, there are about 1.15 billion ducks, and among them, 1.0 billion (88%) are in Asia. In fact, duck production is only second to chickens from the poultry industry (FAO, 2017). Unfortunately, with the rise in duck population in duck farms and slaughterhouses, SE infection has become a widespread challenge (Cha et al., 2013; Yang et al., 2019). In particular, SE developed mechanisms to colonize the avian reproductive tract and, as a consequence, can be vertically transmitted to the offspring *via* transovarian bacterial transmission more effectively than other strains (Gantois et al., 2009). This may lead to persistent infections and difficultly in clearing the poultry flock. As waterfowl, infected ducks are potential reservoirs for SE. This can result in contaminated eggs, vertical transmission, and significant environmental pollution.

SE can utilize fimbriae, flagellae, lipopolysaccharide, and virulence-associated secretion system, encoded by the pathogenicity island, to invade a bird body and cause systemic infections, prior to colonizing the reproductive tract (Gast et al., 2020). The type VI secretion systems (T6SSs) is recognized in numerous bacterial pathogens and is involved in a myriad of activities like biofilm synthesis, interbacterial associations, pathogenesis, cytotoxicity, and phagocytic cell survival (Jana and Salomon, 2019; Schroll et al., 2019; Xian et al., 2020). Moreover, the *Salmonella* Gallinarum or SE T6SS activity was also speculated to regulate various colonization of the digestive tract and other organs of the host range (Blondel et al., 2010). In *Salmonella* Enteritidis, SPI-19 includes some components of the T6SS, one of which corresponds to open reading frame (ORF) SEN1002. This codes for denominated haemolysin co-regulated protein (Hcp), a 28 kDa protein that polymerizes into hexameric rings which forms a tubular structure that is essential for T6SS, permitting the bacteria to secrete effector proteins (Bello et al., 2016). In addition, Hcp, a critical factor in T6SS assembly and intercellular export of its effectors, is known to alter the TNF signaling pathway during SE infection (Zheng et al., 2020). Moreover, a prior investigation revealed that SE possesses certain endogenous properties that facilitate association with either the reproductive organs of egg-laying birds or egg compartments. In our previous study, we demonstrated that the Hcp release and T6SS apparatus stability may be possible candidates for the development of an antimicrobial drug against SE in dGCs, using dual RNA-sequencing (Zhang et al., 2021). Nevertheless, the role of the T6SS component effector protein Hcp in the SE-infected reproductive tract and the reason for the lack of infection response are currently unknown.

Numerous studies revealed that high contamination rates were detected in yolk of eggs (Bichler et al., 1996; Gast and Holt, 2000; Gast et al., 2002). And SE colonizes the ovarian preovulatory follicles by invading and multiplying within the ovarian granulosa cells (GCs) (Thiagarajan et al., 1994; Thiagarajan et al., 1996), suggesting the GCs are the primary SE colonization site within the reproductive tract. The phenomenon of transovarial transmission is an intriguing aspect in the avian pathogenesis of SE. Considering *Salmonella* secretion systems which encoded by pathogenicity islands are essential in the ability to spread within the host and to cause a systemic infection (Jones et al., 2002), herein, we constructed a T6SS core element *hcp* deletion mutant and generated a model of SE infection of dGCs to elucidate the function of Hcp in SE invasion and infection of the reproductive tract, as well as egg contamination in ducks. Our results will provide enhanced comprehension of SE pathogenesis and the subsequent immunologic response in ducks.

## Results

### Construction and the adhesion assay of the mutant MY1△*hcp*

**Figure 1:**
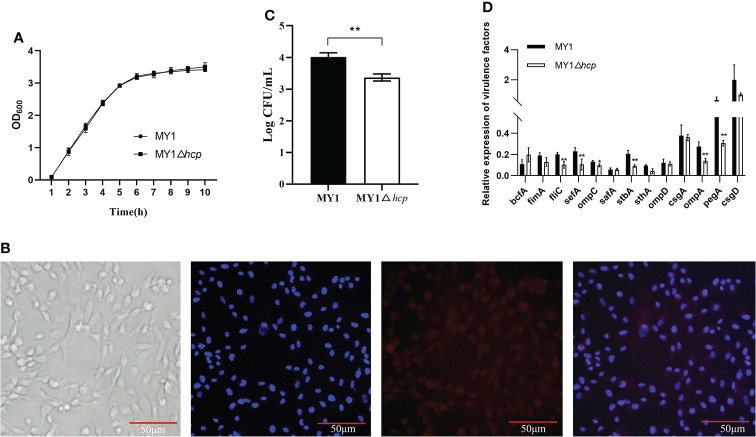
Construction and the invasion assay of the mutant MY1△*hcp*. **(A)** Growth curves of wild-type *Salmonella* Enteritidis MY1 and the mutant MY1△*hcp*. Bacteria were grown in LB at 37°C for 10 h with agitation, and OD_600nm_ values of triplicate cultures were measured every 1 h. Data are the means of three independent experiments. The two-way ANOVA was performed for growth curves (*P* < 0.05). **(B)** Isolation and identification of dGCs. Diagram of a dGC (400×), DAPI staining of cell nucleus, and fluorescent image of FSHR. **(C)** Adherence to dGCs by wild-type *Salmonella* Enteritidis MY1 and the mutant MY1△*hcp*. **(D)** qRT-PCR analysis of the changes of virulence factors expression between MY1 and MY1△*hcp* strain. Data are expressed as mean ± SD of triplicate experiments. **Indicates statistically significant difference compared with the wild-type strain (*P* < 0.01).

Using primers P1 and P2 in PCR amplification, an 1113bp PCR product was identified from the wild type (WT) strain MY1. Then, a 482bp amplified fragment was obtained after the PCR product was electroporated into MY1 containing plasmid pKD46, while the size of the mutant MY1△*hcp* recombinant was 299bp using primers P3 and P4 in PCR amplification (**Figure S1**). Upon sequencing analysis, only the detection FRT site and identification primers P3 and P4 remained in the mutant MY1△*hcp* homologous region. Hence, we successfully constructed an *hcp* mutant within *SE* MY1. As shown in **Figure 1A**, the 10 h growth rates of the *hcp* mutant and parental strain were comparable in LB liquid medium.

Adherence and invasion of SE into duck cells, especially ovarian dGCs, are key mechanisms of successful duck egg contamination and vertical transmission. The isolated dGCs were placed in a monolayer and they adhered successfully to culture flasks in less than 24 h. The dGCs reached 100% confluence and cytoplasmic granulation became apparent within 3 days of culture. DAPI and IFA were employed for dGCs nuclei staining and to identify the cytoplasmic specific receptor FSHRs, respectively (**Figure 1B** **)**. To study whether *hcp* deficiency influences *SE* adhesion, we assessed bacterial interactions and invasion in dGCs *in vitro*. We found that the intracellular MY1△*hcp* numbers plummeted by 15.96%, relative to the number of WT bacteria (**Figure 1C**).

RT-qPCR was used to quantify the expressions of type 1 fimbriae major subunit gene *fimA*, flagella gene *fliC*, SEF14 fimbriae major subunit gene *sefA*, outer membrane protein *ompA*, *ompD*, *ompC*, the major biofilm regulator *csgA*, *csgD*, and other virulence genes such as *bcfA*, *sthA*, *stbA*, *safA*, and *pegA*. The expressions of *fliC*, *sefA*, *ompC*, *stbA*, *ompA*, and *pegA* genes were down-regulated by 49, 54, 26, 56, 49, and 53%, respectively, in the MYΔ*hcp* when compared with their expression in the WT strain. While, there were no differences among all strains in the expression of *bcfA, fimA*, *safA*, *sthA*, *ompD*, *csgA*, and *csgD*. (**Figure 1D**).

### Hcp enhanced SE-mediated apoptosis and immune response in dGCs

**Figure 2:**
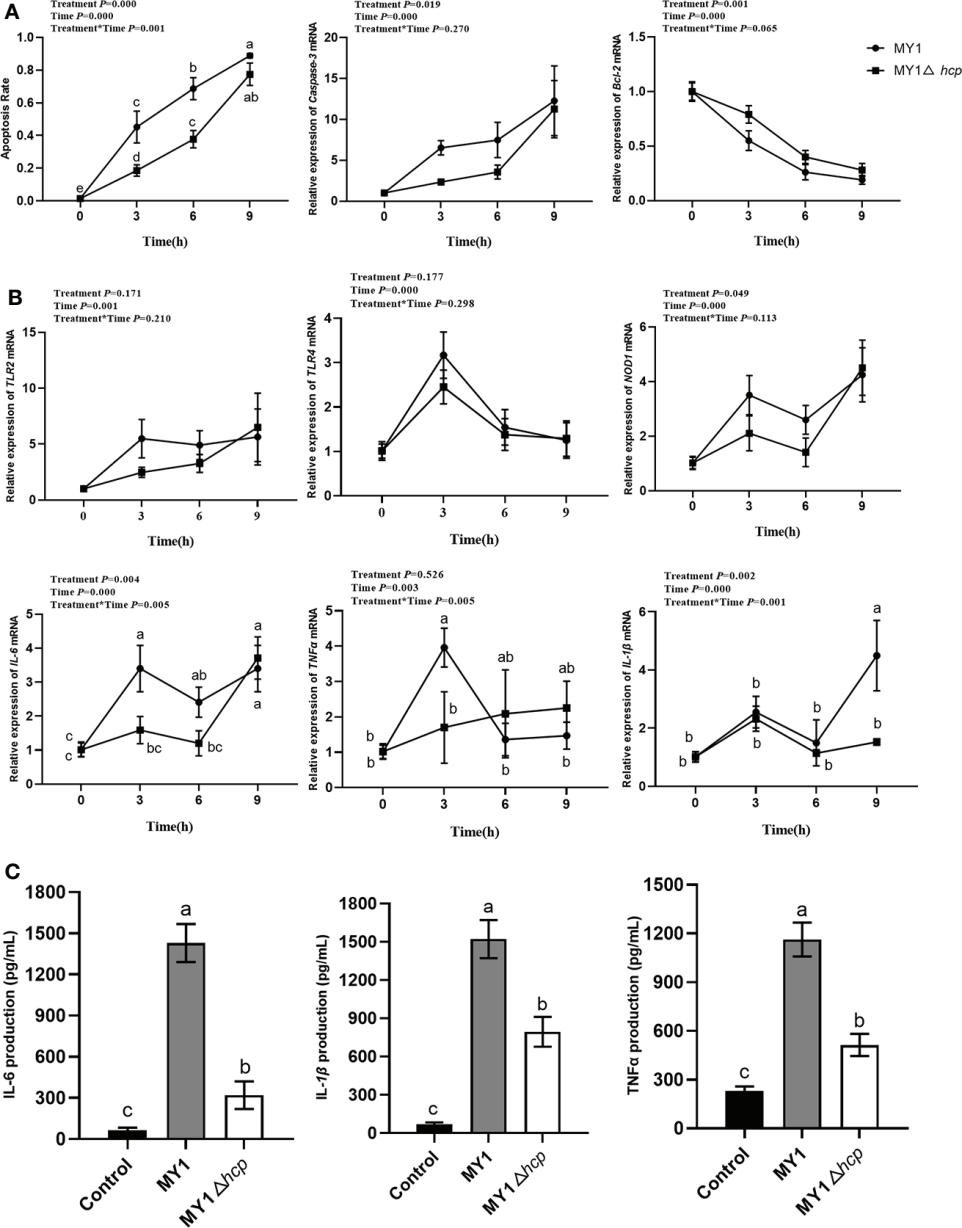
The apoptosis and immune response between MY1-infected and MY1△*hcp*-infected dGCs. **(A)** The dGCs apoptosis rate was detected by Hoechst 33,258 staining and the RT-qRCR results of apoptosis-related gene *Caspase-3* and *Bcl-2*. **(B)** The expression of dGCs immune-response genes *TLR2*, *TLR4*, *NOD1*, *TNFα*, *IL-6*, and *IL-1β* were detected by RT-qRCR. **(C)** The release of proinflammatory cytokines IL-6, IL-1β, and TNF-α release of infected dGCs were detected by ELISA. Data acquired from three individual experiments, and each assay was performed by three biological repetitions. The significant differences were identified using two-way ANOVA analysis, and the mean values ± SD were shown in each plotting. Different superscripts within columns indicate means are significantly different (*P* < 0.05).

To elucidate the Hcp-mediated regulation of dGCs apoptosis during SE challenge, we assessed the infected-dGCs apoptosis rate and detected the expressions of apoptotic genes *Caspase-3* and *Bcl-2*. We observed a decrease in apoptosis in dGCs in the MY1△*hcp*-infected cells, relative to the wild-type strain. The *Caspase-3* levels were markedly elevated in the MY1-infected dGCs, relative to the MY1△*hcp* dGCs, and the anti-apoptotic gene *Bcl-2* showed the opposite result (**Figure 2A**). To further explore the effect of Hcp on host immune responses, the innate immune genes *TLR2*, *TLR4*, *NOD1*, *TNFα*, *IL-6*, *IL-1β* expressions in MY1- and MY1△*hcp*-inoculated dGCs and proinflammatory cytokines IL-6, IL-1β, TNF-α releases were examined by RT-qPCR and ELISA assay, respectively. The results showed that the transcriptions of *TLR2*, *TLR4*, *NOD1*, *TNFα*, *IL-6*, *IL-1β* expressions and IL-6, IL-1β, TNF-α release were strongly augmented in both wild-type and mutant SE-inoculated dGCs. Moreover, the *NOD1*, *TNFα*, and *IL-6* expression levels and IL-6, IL-1β, TNF-α titer indices at 3hpi were markedly downregulated in the MY1△*hcp*-inoculated cells, compared to the MY1-inoculated cells (**Figure 2B**, **C**, **Table S1**).

### Hcp promoted SE-mediated dysregulation of egg production and increase in eggs contamination

**Figure 3:**
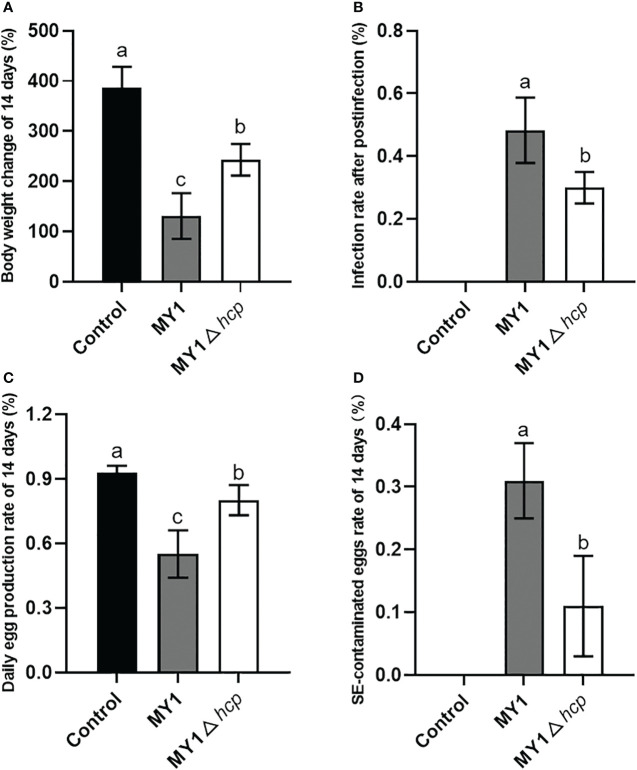
Measurement of body weight change, infection rate, daily egg production and egg contamination between MY1-infected and MY1△*hcp*-infected ducks. **(A)** The body weight change of the control, the MY1-infected and MY1△*hcp*-infected groups of 14 days. **(B)** The infection rate after postinfection between MY1-inoculated and MY1△*hcp*-inoculated ducks (n=3). **(C)** The daily egg production of the control, the MY1-infected and MY1△*hcp*-infected groups of 14 days. **(D)** The egg contamination rate of the control, the MY1-infected and MY1△*hcp*-infected groups of 14 days. Significant differences of daily egg production and egg contamination rate results were identified using one-way ANOVA statistical analysis (n=14). Different superscripts within columns indicate means are significantly different (*P* < 0.05).

Following 3 days post-infection, 8 MY1-inoculated and 6 MY1△*hcp*-inoculated ducks were obtained, and assessed for daily egg output and egg contamination over 14 days. These 14 infected ducks exhibited persistent diarrhea throughout the study. There were significant differences in the body weight and infection rate after postinfection between MY1-inoculated and MY1△*hcp*-inoculated ducks (**Figures 3A**, **B**). To determine the role of Hcp on egg output and egg contamination during SE infection, we monitored the daily egg output and egg contamination rate of each group. The results showed that MY1-inoculated ducks produced the lowest yields of eggs, with the highest contamination. In contrast, the MY1△*hcp*-inoculated ducks had more egg yield and fewer contaminations, relative to the MY1-inoculated ducks (**Figures 3C**, **D** **)**.

### Quantitative proteomics analysis between the MY1- and MY1△*hcp*-infected dGCs

We employed label-free differential proteomic analyses to establish a comprehensive understanding of the proteome dynamics and alteration of host responses related to the bacterial effector Hcp. A quantitative proteomics analysis between MY1- and MY1△*hcp*-infected infected dGCs at 3hpi. We identified a total of 1,579,224 spectrums (MS/MS spectra), 57, 686 peptides, and 5,387 proteins (**Table S2** **)**. The identified proteins were distributed in each molecular weight segment, and the sequencing results were of high quality (**Figures S2A–C**). In all, there were 4,098 (76.07%, 4,098/5,387) proteins, and 2,709 (50.29%, 2,709/5,387) annotated into the GO and KEGG databases. We performed PCA and RSD clustering to validate sample distribution and biological reproducibility, respectively (**Figures S2D–F**).

**Figure 4:**
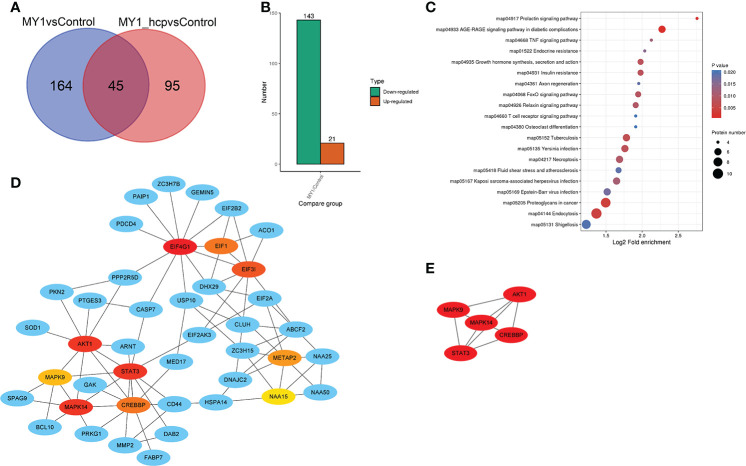
Differentially abundant proteins (DAPs) analysis between MY1-infected and MY1△*hcp*-infected dGCs. **(A)** Venn diagram of pairwise comparison of MY1-infected and MY1△*hcp*-infected dGCs sequencing samples. **(B)** Statistics of the number of DAPs. **(C)** Kyoto Encyclopedia of Genes and Genomes **(KEGG)** pathway analysis for the DAPs. *P* < 0.05 was used as a threshold to select significant KEGG pathways. **(D)** The protein protein interaction (PPI) network of DEPs of top 10 with first-stage neighbors. **(E)** According to cytoHubba plugin’s MCC ranking, the five hub DAPs the five hub DAPs of the PPI network.

**Figure 4A** illustrates the differentially abundant proteins (DAPs) between the two groups after data adjustment as follows, (*P* < 0.05 and Fold Change > 1.5 or < 0.67). Among the 164 DAPs associated with Hcp infection, 143 were highly up-regulated and 21 were significantly down-regulated (**Figure 4B**). We next performed KEGG pathway enrichment classification (**Table S3** **)**. The 20 leading KEGG pathways associated with DAPs were as follows (**Figure 4C** **)**, prolactin; TNF; growth hormone synthesis, secretion and action; FoxO, T cell receptor; necroptosis; endocytosis; and so on. Collectively, these pathways play key roles in immunity, hormone synthesis, cell proliferation, and cell apoptosis. Proteins generally interact with other proteins in biological functions, and, therefore, the protein-protein interactions of DAPs were next analyzed. According to the Cytoscape cytoHubba plugin’s MCC ranking, the key interaction network and top five hub differential proteins were STAT3, AKT1, MAPK9, MAPK14, and CREBBP, which were located at the center of the entire regulatory network (**Figures 4D**, **E**). Hence, we speculated that these host proteins and their related functions were the most significantly affected in relation to bacterial Hcp.

### Parallel reaction monitoring (PRM) analyses of DAPs in the MY1- and MY1△*hcp*-infected dGCs

**Figure 5:**
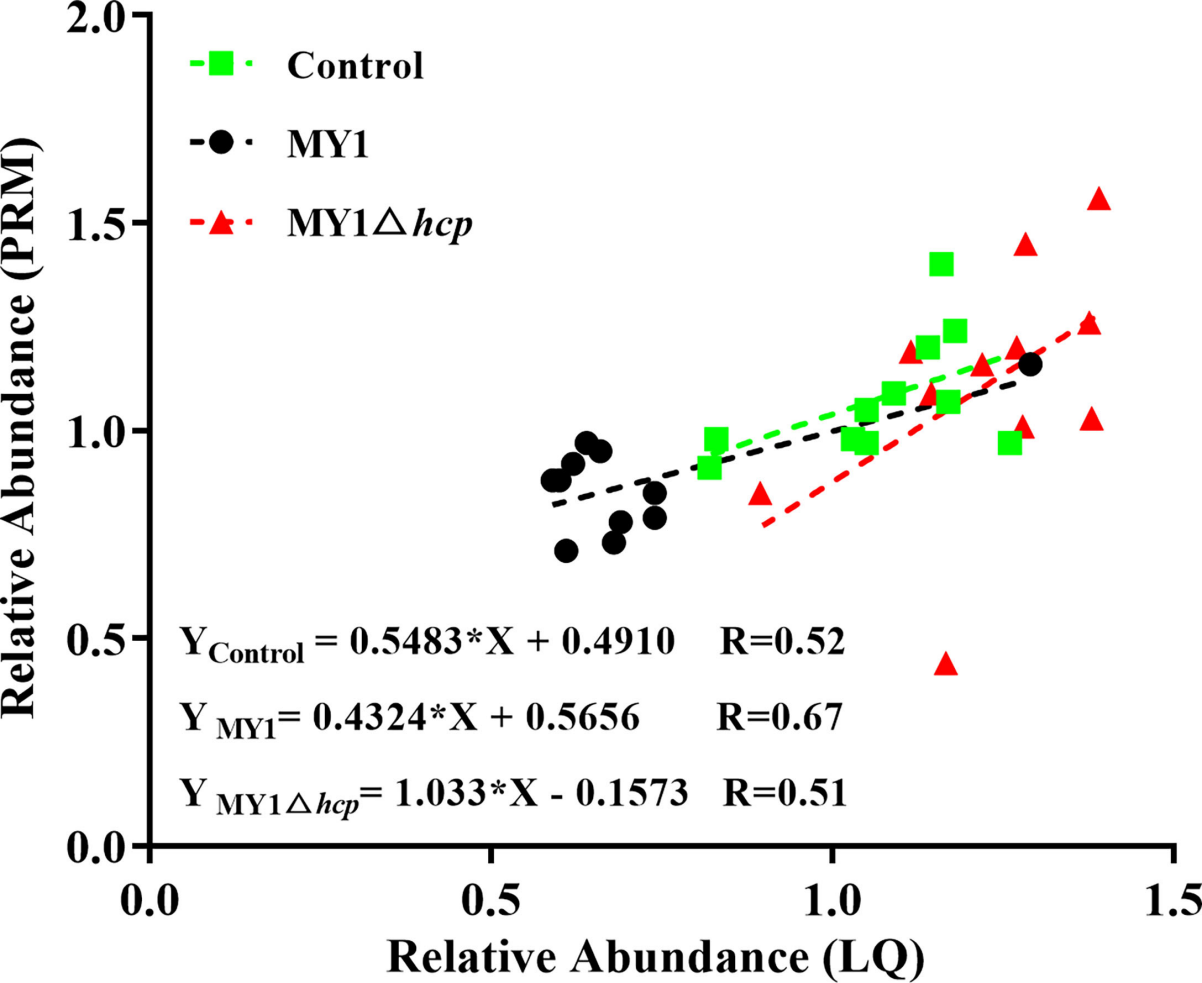
Linear regression fitted for relative abundance of selected proteins determined *via* LQ and PRM. The selected proteins in samples were used for linear regression analysis.

We next employed PRM to validate the levels of 11 LC-MS/MS-scanned proteins, namely, STAT3, BTF3, CREBBP, AKT1, PKN2, DOCK1, NEK7, CASP7, MAPK9, TP53I3, and MAPK14 using non-targeted proteomics. Based on our results, the PRM data was successful in characterizing host DAPs, as it was comparable to our quantitative proteomics analysis, thereby validating our proteomics analysis (**Figure 5**, **Table S4** and **Figure S3**).

## Discussion

T6SS is a newly discovered secretory system that is common among Gram-negative bacteria. *Salmonella* T6SS has the potential to enhance virulence and environmental adaptability of pathogens, thus providing bacteria with selective advantages and abilities to survive and proliferate in host cells (Blondel et al., 2015; Sana et al., 2016; Troxell, 2018). Hcp is the hallmark and core component of T6SS. It is a structural protein that can serve as a secretory or chaperone protein, and it promotes bacterial invasion of host tissue, host immune response escape, and persistence within the host cell (Feria and Valvano, 2020). Moreover, numerous studies revealed that Hcp in bacteria like *Pseudomonas aeruginosa*, *Burkholderia pseudomallei*, and *Salmonella* Typhimurium contribute to bacterial virulence, pathology, and intracellular growth within macrophages (Lim et al., 2015; Wang et al., 2020; Howard et al., 2021). Besides, Hcp was shown to elicit immune responses in mice, horses, and humans, and was recognized as an interesting candidate for vaccine design (Bello et al., 2016). Thus, it is crucial to examine the pathogenesis of T6SS and provide strategies for its elimination. Herein, we generated an *hcp* deletion mutant MY1△*hcp* and examined infection characteristics *in vitro*. We demonstrated that the SE invasion and adhesion abilities decreased by 15.96% and the apoptotic rate decreased in infected dGCs, upon deletion of *hcp*. Adherence is a critical prerequisite event for pathogen infection. It was reported that *hcp* contributes to the adhesion of *Escherichia coli* and *Vibrio parahaemolyticus* to host cells (Zhou et al., 2012; Yu et al., 2012; Ding et al., 2018). In the present study, the *hcp* has significant regulation on the expression of adhesion-related genes such as *sefA*, *ompC*, *stbA*, *ompA*, and *pegA*, also indicating that *hcp* is important in SE adhesion. Moreover, Hcp contributes to the pathogenesis of *Salmonella* Typhimurium, during the late stages of macrophage infection (Mulder et al., 2012). Likewise, Suarez *et al.* also reported that *Aeromonas hydrophila* Hcp translocates to the host cytoplasm, whereby it induces apoptosis by activating *caspase-3* (Suarez et al., 2008). These evidences suggest that a potent Hcp-mediated cell adhesion and invasion activity facilitates SE pathogenesis.

SE infection delays steroid hormone production and impairs cell growth in hen GCs. Identification of altered genes, using microarrays, revealed genes regulating host immunity, physiological activities, signaling pathways, and transcription of genes (Tsai et al., 2010; Wang et al., 2014). Herein, we demonstrated that the levels of immunogenic genes *TLR4*, *NOD1*, *TNF-α, IL-1β* and proinflammatory cytokines IL-6, IL-1β, TNF-α were markedly downregulated in the MY1△*hcp*-inoculated cells, as opposed to the WT MY1 strain. We also noted that DAPs between the MY1- and MY1△*hcp*-infected dGCs were enriched in the TNF, prolactin, growth hormone synthesis, secretory and action pathways, etc. Similar to our report, Zheng et al. also showed that SE Hcp regulates levels of the TNF, IL-17, and cytokine-cytokine receptor interaction pathways in BHK-21 cells (Zheng et al., 2020). Hcp is also known to interact with macrophages to promote synthesis of immunosuppressive cytokines like IL-10 and TGF-β, which paralyzes macrophages and prevents phagocytosis (Suarez et al., 2010). Based on these evidences, Hcp is intricately linked to the bacteria-host interaction. Moreover, this interaction not only involves the reproductive tract local immune response, but also affects host physiology as a whole.

We next analyzed the protein-protein interaction network even further and demonstrated that the infection related proteins STAT3, AKT1, MAPK9, MAPK14, and CREBBP had the highest degree of connectivity among DAPs. It was reported both *in vivo* and *in vitro* that STAT3 is activated early after WT *Salmonella* infection (Lin and Bost, 2004). Hannemann *et al.* demonstrated that *Salmonella* Typhimurium stimulates STAT3 *via* an non-traditional network, that excludes JAK kinases, and is initiated by SPI-1 T3SS effectors (Hannemann et al., 2013). Moreover, *Salmonella* Typhimurium is suggested to employ a single effector, SopB, to regulate a wide variety of host kinases to activate Akt for optimal bacterial replication within host. These evidences suggest that STAT3, AKT1, MAPK9, MAPK14, and CREBBP are the main genes that respond to Hcp during *SE* infection.

Prior studies revealed that SE has some endogenous properties that facilitate a specific host association that results in chronic colonization and reproductive organs contamination (Gantois et al., 2009). Reproductive tract contamination is the product of systemic infection (Wigley, 2014). Hsieh et al. discovered the Hcp-regulated antibacterial function of T6SSs offers a superior edge to *Klebsiella pneumoniae* during intestinal infection and extensive dissemination in mice (Hsieh et al., 2019). Using functional analyses, it was revealed that Hcp in *Campylobacterjejuni* regulates colonization *in vivo* by augmenting adhesion and invasion into host eukaryotes, thus resisting bile salts and deoxycholic acids (Noreen et al., 2018). Herein, we also demonstrated that Hcp aids in SE invasion and multiplication within ovarian GCs, which results in egg contamination and reduced egg production in ducks. Hence, we believe Hcp enhances pathogenicity and promotes transovarian transmission of SE in laying ducks. Our results demonstrated interactions between bacterial effector and host cell proteins during ovarian transmission, offering a novel candidate subunit for the development of vaccine against SE.

## Conclusion

In summary, we identified that the bacterial Hcp accelerates SE invasion of ovarian GCs, induces cell apoptosis, and suppresses immune related genes like *TLR4*, *NOD1*, *TNFα*, and *IL-1β* expression and proinflammatory cytokines IL-6, IL-1β, TNF-α release. We also identified an interaction network of 164 DAPs and core responding proteins like STAT3, AKT1, MAPK9, MAPK14, and CREBBP between the bacterial Hcp and host dGCs during SE infection. These findings add to the existing knowledge of SE pathogenesis and related host infection responses in ducks, and offer a novel candidate subunit for the development of vaccine against SE.

## Materials and methods

### Ethics statement

All protocols concerning animals received ethical approval from the appropriate boards at Yangzhou University (Jiangsu, China). The ducks were maintained in standard housing, as described in the publication of Laboratory Animal Requirements of Environment and Housing Facilities (GB 14925-2001).

### Bacterial strains and construction of the Hcp deletion mutant

Bacteria and plasmids used in this study are listed in **Table 1**. The SE strain MY1 and MY1△*hcp* were grown in Luria-Bertani broth (LB) at 37°C. The recombined strains, carrying the temperature-responsive plasmids pCP20 or pKD46, were grown under the same conditions at 30°C. The strains carrying the antibiotic resistance were maintained in LB with 100μg/ml of Ampicillin (Amp) or 34μg/ml of chloramphenicol (Cm), as needed.

**Table 1:** Bacterial strains and plasmids used in this study.

Strains/plasmids	Characteristics	References
Strains		
CMCC(B)MY1	*Salmonella* enterica serovar Enteritidis wild type	NICPBP, China
MY1△*hcp*	*hcp*-deficient mutant	This study
Plasmids		
pKD3	Cm^r^; Cm cassette template	Datsenko & Wanner 2000
pKD46	Amp^r^, λRed recombinase expression	Datsenko & Wanner 2000
pCP20	Amp^r^, Cm^r^; Flp recombinase expression	Datsenko & Wanner 2000
pMD19 T-simple	Cloning vector, Amp^r^	Takara

The *hcp* deletion mutant was synthesized with the λ-Red recombinase-mediated recombination system as described previously (Datsenko and Wanner, 2000; Duan et al., 2013). The primers are summarized in **Table 2**. In short, primers P1 and P2 were used to increase the yield of chloramphenicol cassette of pKD3 plasmid. This included the 48-bp homology extensions from the 5’ and 3’ of *hcp*. We next purified the PCR products, prior to electroporation into pKD46 carrying MY1. The recombinant bacteria MY1△*hcp*::cat was identified and isolated on both Cm and Amp resistance LB agar plates. The allelic *hcp* replacement *via* the Cm cassette (CmC) was further confirmed using PCR assay, employing primers P3 and P4, prior to DNA sequencing. Subsequently, the CmC gene of MY1△*hcp*::cat was isolated *via* inclusion of the Flp recombinase-harboring vector pCP20. Thus, the entire *hcp* deletion mutant was achieved, and verified using PCR and DNA sequencing. Lastly, growth rate determination of MY1 and MY1△*hcp* were done by culturing them with agitation (180 rpm/min) in liquid LB at 37°C and simultaneously measuring OD_600_ nm at the end of each hour. The growth curve assay was performed in three individual experiments.

**Table 2:** Primers used for qRT-PCR analysis of the changes of virulence factors expression between MY1 and MY1△*hcp* strain.

Primer	Sequence (5′–3′)	Size (bp)
*fimA*	F: 5′-GACTGCGATCCGAAAGTGG-3′ R 5′-CAGAGGAGACAGCCAGCAAA-3′	91
*fliC*	F 5′-ATTGAGCGTCTGTCCTCTGG-3′ R 5′-GATTTCATTCAGCGCACCTT-3′	170
*sefA*	F 5′-TGCTGCTGGTCAGAAAGTTG-3′ R 5′-TATTGGCTCCCTGAATACGC-3′	167
*bcfA*	F 5′-TGACGCTGCCTGTTCTGTTT-3′ R 5′-GCAGTCTTCCAGTTTGATGGTG-3′	136
*sthA*	F 5′-AAGAAATCCGTGTTGGTCGT-3′ R 5′-GGCAACCGATAACCATGACT-3′	165
*stbA*	F 5′-ATCACAGGCTCGCTTCTTGT-3′ R 5′-CGGACAGCCCAATATCAAAC-3′	211
*safA*	F 5′-GGTTGCTAACACGACACTGG-3′ R 5′-CAAAGGTGAACCAGCTCCTC-3′	152
*ompC*	F 5′-AAAGTTCTGCGCTTTGTTGG-3′ R 5′-CGCTGACGAACACCTGTATG-3′	162
*ompD*	F 5′-ACGGTCAGACTTCGCATAGG-3′ R 5′-TGTTGCCACCTACCGTAACA-3′	185
*ompA*	F 5′-ACTGAACGCCCTGAGCTTTA-3′ R 5′-ACACCGGCTTCATTCACAAT-3′	135
*csgA*	F 5′-ATGCCCGTAAATCTGAAACG-3′ R 5′-CCGTATTGGCCGACAGTAAT-3′	172
*csgD*	F 5′-GCCTCATATTAACGGCGTGT-3′ R 5′-GGACTCGGTGCTGTTGTAGC-3′	157
*pegA*	F 5′-GAAGTGGTGGGAACATCCTG-3′ R 5′-GAAGCCCGCACCATTATTAG-3′	249
*gyrA*	F 5′-GCATGACTTCGTCAGAACCA-3′ R 5′-GGTCTATCAGTTGCCGGAAG-3′	278

### Experimental infection of animals

*Salmonella*-free, 26-week-old female Shaoxing ducks were acquired from the National Waterfowl Conservation Center (Jiangsu, China). The control and experimental animals were maintained in distinct pens, but under same conditions. Our infection model was as reported earlier (Deng et al., 2008; Zhang et al., 2019). The bacterial load was determined *via* pilot experimentation. In brief, ducks (n = 20, each) were intravenously injected with 10^8^ CFU SE MY1 or MY1△*hcp* (experimental group), respectively, whereas, control ducks (n = 20) were given the same amount of PBS. Subsequently, 3 days post-inoculation, the IDEXX SE Ab examination was employed to measure serum SE antibodies. The infected ducks were maintained in individual cages, contaminated eggs were accumulated, and egg production yield and infection (PCR detection of specific SE gene *sdf*1) were assessed over for 14 days (Agron et al., 2001). The body weight change, infection rate after postinfection, and egg production rate were counted. The infection rate after postinfection represents the percent of total antibody positive individuals from each group (n=3). The egg production rate means the percent of eggs produced in each group (total number eggs/total number ducks) per day (n=14). The egg contamination rate means the percent of total number of infected eggs from each group (total number of infected eggs/total number of eggs) per day (n=14).

### dGC isolation and SE infection assay

dGCs were harvested and grown as described previously (Gilbert et al., 1977; Zhang et al., 2019). In short, adult prehierarchical follicles were obtained from egg-laying ducks. The follicles were next washed with PBS to eliminate yolks and vitelline membranes. Following this, tissues were chopped into 1–2 mm^3^ sections and treated with type II collagenase (Sigma, MO, United States) for 5 min at 37°C to initiate digestion. The resulting suspension was filtered using a 200-μm nylon filter, spun two times at 67 × g for 5 min, rinsed again in M199 media to eliminate collagenase and cell debris, then resuspended in 3 mL of 50% Percoll, prior to spinning at 421 × g for 15 min, and subsequent cell layer isolation. The dGCs were next mixed in M199 media. Cells were maintained in tissue culture flasks for 24 h for adherence prior to experimentation. Purity was assessed *via* indirect immunofluorescence assay (IFA), and follicle GC-specific receptor, as well as FSHR expressions, were assessed with anti-FSHR antibodies (1:500, Proteintech, IL, United States).

Bacterial adherence and invasion were assessed as reporter earlier (Gilbert et al., 1977; Meng et al., 2019). In short, bacteria were cultured till OD_600nm_ of 2.0 in LB broth at 37°C. dGCs (1 × 10^5^ cells per well) were grown in 96-well plates for 24 h, prior to three rinses with sterile PBS (pH 7.2). Next, dGCs were maintained in 100 μl bacterial suspension in Dulbecco’s modified Eagle’s medium for 2 hr at 37°C, with a multiplicity of infection (MOI) of 10 in each well. Following a 2 hr of culture, the inoculated cells were rinsed thrice with PBS to eliminate loosely adherent bacteria. The bacteria cultures were diluted serially in PBS (0, 10, 100, and 1,000) and plated 100μL suspension onto LB agar plates to enumerate adherent bacteria for colony forming unit (CFU) number. These experiments were performed in triplicate with three biological repetitions. The bacteria RNA extraction and qRT-PCR amplification of the virulence factors was performed as described previously (Ding et al., 2018). The primers are list in **Table 2**. The assay were performed in triplicates and the data were normalized to the endogenous *gyrA* level. To assess cell apoptosis following SE infection, the infected cells were stained with Hoechst 33258 at 3, 6, 9 hpi before incubation for 10 min (Crowley et al., 2016). Apoptotic cells, displaying thick and broken nuclei, were monitored under a fluorescence microscope accompanied with a Leica DMIL LED and digital camera ICMOS HD camera. **Table 3** lists the duck immune response-specific primers used in qRT-PCR. The qRT-PCR was carried out with the ABI PRISM 7500 HT system (Applied Biosystems, Carlsbad, CA, USA). Relative transcript levels were determined by the 2-^ΔΔ^Ct formula, followed by adjustment with the endogenous *NADPH* levels. These experiments were performed in triplicates with three biological repetitions.

**Table 3:** Primers used for qRT-PCR analysis of apoptosis and immune response genes during *Salmonella* entertidis infection.

Primer	Sequence (5′–3′)	Accession number	Size (bp)
P1-2	F:5′-CAGTGTTCCCAGTTTATGGATTCATATATAAAAGAAATACTTTCCTGACATATGAATATCCTCCTTAG-3′ R:5′-TTTCAGCAGGTTGTTCCCTTGACTCTATCGGTAATAAAGCACAGTTATGTGTAGGCTGGAGCTGCTTCG-3′		1113
P3-4	F: 5′-TACGGTACCCTGAAGCGACACATTC-3′ R: 5′-CGCCTCGAGTACTTTCATCGTTCAT-3′	NC_011294.1	482
*Caspase-3*	F: 5′-TAGCAGGAAAACCCAAAC-3′ R: 5′-AGACTGAATAAACCAGGAGC-3′	XM_027456288.2	200
*Bcl-2*	F: 5′-ACTTCATCAAGATTGCCTCC-3′ R: 5′-CTGTTATGCCGTGCTGGT-3′	XM_038173909.1	117
*TLR2*	F: 5′-CACTTCCGCCTATTTGACGAGA-3′	KX687002	115
	R: 5′-TTGTGTTCATTATCTTCCGCAGT-3′		
*TLR4*	F: 5′-ATAAAAGAACTGGTCGAACCC-3′ R: 5′-TGCTCTCCAGAAAGTCGGTA-3′	NM_001310413	169
*NOD1*	F: 5′ GTGACTTTCTTGGGCTTATACAACA 3′ R: 5′ AGGCACTTCCCTCCTTCGCTA 3′	NM_001310381	140
*TNFα*	F: 5′ GATGGGAAGGGGATGAAC 3′ R: 5′ ATTACAGGAAGGGCAACA 3′	XM_021277517	144
*IL-6*	F: 5′ AAAGCATCTGGCAACGAC 3′ R: 5′ GAGGAGGGATTTCTGGGT 3′	JQ728554	88
*IL-1β*	F: 5′-CCGAGGAGCAGGGACTTT-3′ R: 5′-AGGACTGTGAGCGGGTGTAG-3′	DQ393268	133
*GAPDH*	F: 5′-TGCTAAGCGTGTCATCATCT-3′ R: 5′-AGTGGTCATAAGACCCTCCA-3′	XM_038180584.1	60
*Sdf1*	F: 5’-GAATCAGTATAATTCGTCAATACCTAAG-3’ R: 5’-ATTCAATTTCTGTCGCATATATGCTTAA-3’	GD165044.1	293

The monolayer cells were incubated with bacteria at an MOI of 10 with 2hrs. Then the supernatant of infected cells was harvested at 3 hpi. The concentrations of cytokines TNF-α, IL-6, and IL-1β in the supernatant of dGCs cells were measured using the corresponding cytokine ELISA kits (Jiancheng Bio, Nanjing, China) according to the kit manual. Data acquired from three individual experiments, and each assay was performed by three biological repetitions..

### Label-free quantitative proteomics of MY1- and MY1△*hcp*-inoculated dGCs

Nine 3hpi samples, namely, MY1-; MY1△*hcp*- and non-infected cells (n=3, respectively), were used to conduct quantitative proteomics of dGCs. Briefly, we introduced four times the lysis buffer to the cells, prior to sonification, then centrifuged at 12,000g for 10 min at 4°C, to eliminate cellular debris. The resulting supernatant was collected into a fresh tube, followed by protein quantification using a bicinchoninic acid (BCA) kit, as per kit directions.

To conduct trypsin-mediated digestion, the protein underwent reduction with 5 mM dithiothreitol for 30 min at 56°C. The protein sample was next diluted till a uric concentration of <2 M. Then, trypsin was introduced at a 1:50 trypsin-to-protein mass ratio to initiate overnight (O/N) digestion. Additional trypsin was introduced at a 1:100 ratio for a secondary 4 h digestion. The resulting peptides were resuspended in mobile phase A (MPA) for liquid chromatography (LC) analysis, followed by separation with the NanoElute ultrahigh performance liquid system (UHPLS). The aqueous MPA consisted of 0.1% formic acid (FA), whereas, mobile phase B consisted of acetonitrile with 0.1% FA. The liquid gradient was set at: 0-18 min, 6-24% B; 18-24 min, 24-32% B; 24-27 min, 32-80% B; and 27-30 min, 80% B. The flow rate was kept steady at 450 nL/min.

Peptides separated by UHPLS were inserted into the capillary ion source for ionization before analysis with a timsTOF Pro mass spectrometer. The secondary mass spectrum (MS) scanning range was adjusted to 100–1700 m/z., the parallel accumulation serial fragmentation (PASEF) mode was employed for data acquisition. Upon collection of the first-level MS, the second-level MS with parention charges in the range of 0–5 were recorded 10 times in the PASEF mode.

The secondary MS data were extracted with Maxquant (v1.6.6.0). The *Anas platyrhynchos* 8839_TX_20200225.fasta (23317 sequence) database (Uniprot: https://www.uniprot.org/taxonomy/8839). The parameters were set as follows: enzyme digestion employed trypsin/P; the missing cleavage site number was adjusted to 2. In terms of protein and peptide spectral match identifications, the FDR was adjusted to 1%.

The recognized proteins were stratified and clustered into distinct networks *via* the KEGG pathways (Version 70). The STRING 10 database (http://string.embl.de/) was employed for protein-protein association analysis.

### 4D PRM-based result validation

We used 100 mM TEABC to dilute 3hpi cell samples (n=3, each group) and the proteins were reduced with 10 mM DTT, prior to alkylation with 20 mM IAA. Following this, proteins were double digested with Lys-C and trypsin, prior to peptide purification in Sep-Pak column C18 material and drying of samples using a speed vac.

The digested peptides were resuspended in 0.1% FA and injected into a trap column (100 µm × 2 cm, Nanoviper). To conduct PRM analysis, 5 µl unfractionated CSF peptides were dissociated on an analytical column (75 µm × 50 cm, RSLC C18). The employed linear gradient ranged between 4-35% buffer B over 75 min. Every target protein was monitored using several peptides from a target inclusion list. The PRM scan was adjusted to: 35,000 orbitrap resolution, 5 x 10^5^AGC target value, 100 ms injection duration, 2 m/z isolation duration, 27 HCD normalized collision energy, and m/z 110 starting mass.

The PRM data was next compared using the Skyline package (Maclean et al., 2010). Peak integration was carried out within the platform and evaluated manually. The data were then extracted for further information and analyses.

### Statistical analyses

Data are means-standard deviation (SD), and experiments were repeated at least three times. Data were analyzed with SPSS 26.0 (Chicago, IL, United States) *via* one-way ANOVAs with Dunnett’s test, with *p*-value <0.05 as the significance threshold. Turkey-HSD multiple range test was used to analyze the main effect among different treatments and different time for the data of apoptosis rate, apoptosis-related gene and immune-response gene expression levels from RT-qPCR.The TMT data was analyzed using the Perseus software package (Tyanova et al., 2016). Students’ t-test was employed to compare between groups. Proteins with *p*-value <0.05 were deemed as significant in AD. Logistic regression and ROC curve analyses for select markers and in combinations were performed *via* the R package (Robin et al., 2011).

### Data Access

The protein sequencing data can be accessed through PRIDE database (http://www.ebi.ac.uk/pride) with accession number PXD032735.

## Data availability statement

The datasets presented in this study can be found in online repositories. The names of the repository/repositories and accession number(s) can be found in the article/**supplementary material**.

## Ethics statement

The animal study was reviewed and approved by The Institutional Animal Care and Use Committee of Yangzhou University (Jiangsu, China).

## Author contributions

YZ and QX conceived and designed the experiments. GZ and GC assisted in experimental design. LS and JW performed the experiments. KW, FY, and WV analyzed the data. YZ and LS wrote the manuscript. All authors contributed to the article and approved the submitted version.

## Funding

This study was supported by the National Natural Science Foundation of China (31702107) and the Open Project Program of Joint International Research Laboratory of Agriculture and A Project Funded by the Priority Academic Program Development of Jiangsu Higher Education Institutions (PAPD).

## Conflict of interest

The authors declare that the research was conducted in the absence of any commercial or financial relationships that could be construed as a potential conflict of interest.

## Publisher’s note

All claims expressed in this article are solely those of the authors and do not necessarily represent those of their affiliated organizations, or those of the publisher, the editors and the reviewers. Any product that may be evaluated in this article, or claim that may be made by its manufacturer, is not guaranteed or endorsed by the publisher.

## Supplementary material

Supplementary Figure S1 Identification of MY1△*hcp* mutants by PCR. M: DL2000 marker, 1: PCR products of combination of *cat* gene in plasmid pKD3 with *hcp* homology extension, 2: MY1, 3: MY1△*hcp*::cat, 4: MY1△*hcp*.

Supplementary Figure S2 Quality control of MY1-infected and MY1△*hcp*-infected dGCs proteome. **(A)** The distribution of Peptide length; **(B)** The distribution of peptide number; **(C)** The distribution of protein molecular weight; **(D)** Protein function annotation; **(E)** Principal component analysis (PCA) to evaluate protein quantitative repeatability; **(F)** Relative standard deviation (RSD) to evaluate protein quantitative repeatability.

Supplementary Figure S3 Peptide fragment ion peak area distribution of 11 differentially abundance proteins.

Supplementary Table S1 The apoptosis and immune response between MY1-infected and MY1△*hcp*-infected dGCs.

Supplementary Table S2 Basic information and statistics of MY1-infected and MY1△*hcp*-infected dGCs proteome.

Supplementary Table S3 The statistics of differentially abundance proteins by proteomics between MY1-infected and MY1△*hcp*-infected dGCs.

Supplementary Table S4 Quantitative verification of key proteins by parallel reaction monitoring technology (PRM).
