# Supplementary figures and images for: The salmonella effector Hcp modulates infection response, and affects salmonella adhesion and egg contamination incidences in ducks
Source: Front Cell Infect Microbiol. 2022 Oct 3;12:948237. doi: 10.3389/fcimb.2022.948237 (PMC9575552; doi:10.3389/fcimb.2022.948237)

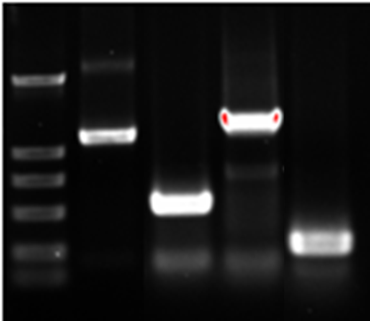

Supplement: Supplementary Figure S1 — Identification of MY1△hcp mutants by PCR. M: DL2000 marker, 1: PCR products of combination of cat gene in plasmid pKD3 with hcp homology extension, 2: MY1, 3: MY1△hcp::cat, 4: MY1△hcp. [file Image_1.tif]

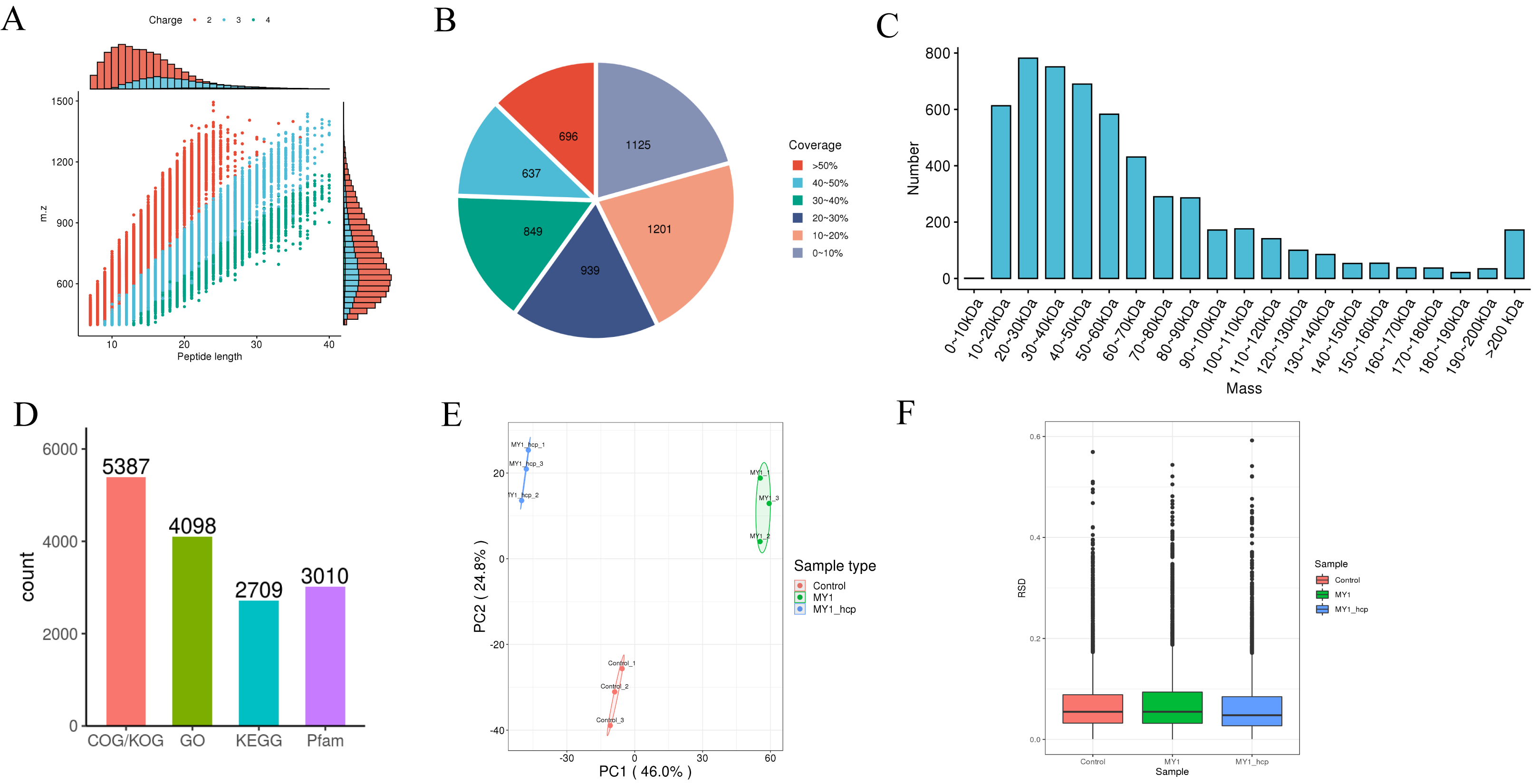

Supplement: Supplementary Figure S2 — Quality control of MY1-infected and MY1△hcp-infected dGCs proteome. (A) The distribution of Peptide length; (B) The distribution of peptide number; (C) The distribution of protein molecular weight; (D) Protein function annotation; (E) Principal component analysis (PCA) to evaluate protein quantitative repeatability; (F) Relative standard deviation (RSD) to evaluate protein quantitative repeatability. [file Image_2.tif]

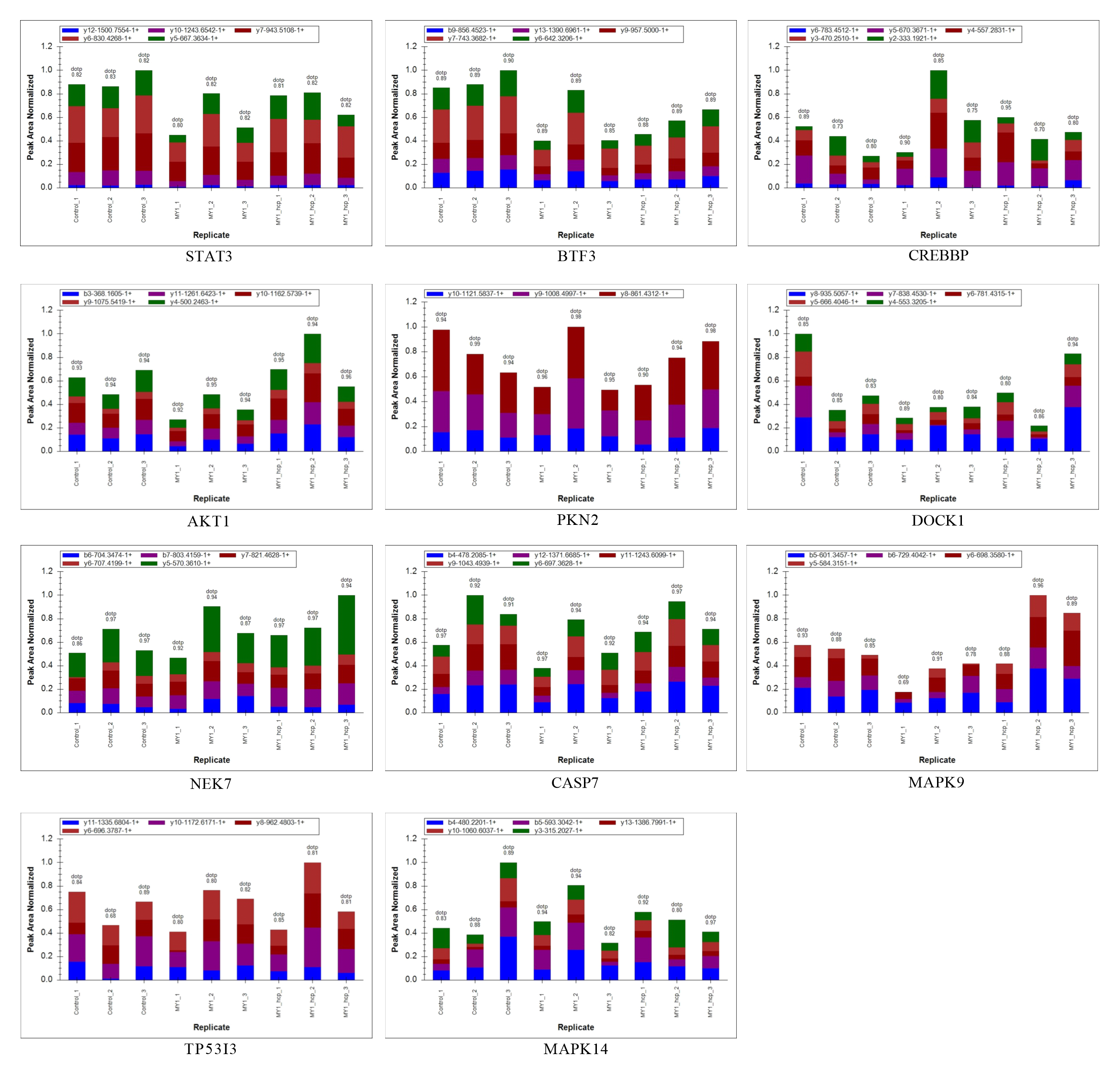

Supplement: Supplementary Figure S3 — Peptide fragment ion peak area distribution of 11 differentially abundance proteins. [file Image_3.tif]
