# Supplementary material for: The salmonella effector Hcp modulates infection response, and affects salmonella adhesion and egg contamination incidences in ducks
Source: Front Cell Infect Microbiol. 2022 Oct 3;12:948237. doi: 10.3389/fcimb.2022.948237 (PMC9575552; doi:10.3389/fcimb.2022.948237)
Supplement: Supplementary Table S1 — The apoptosis and immune response between MY1-infected and MY1△hcp-infected dGCs. [file Table_1.docx]

| Item | | Apoptosis Rate | Relative expression of apoptosis-related genes (2-ΔΔCt) | | Relative expression of immune-response genes (2-ΔΔCt) | | | | | |
| --- | --- | --- | --- | --- | --- | --- | --- | --- | --- | --- |
|  |  |  | *Caspase-3* | *Bcl-2* | *TLR2* | *TLR4* | *NOD1* | *IL-6* | *TNFα* | *IL-1β* |
| 0 hpi | MY1 | 0.01±0.00^e^ | 1.00±0.03 | 1.00±0.09 | 1.00±0.11 | 1.01±0.16 | 1.02±0.24 | 1.01±0.20^c^ | 1.01±0.19^b^ | 1.01±0.18^b^ |
|  | MY1△*hcp* | 0.01±0.00^e^ | 1.00±0.10 | 1.00±0.08 | 1.01±0.12 | 1.02±0.21 | 1.02±0.21 | 1.02±0.22^c^ | 1.02±0.22^b^ | 1.01±0.07^b^ |
| 3 hpi | MY1 | 0.45±0.01^c^ | 6.53±0.87 | 0.55±0.09 | 2.47±0.44 | 2.45±0.38 | 2.11±0.64 | 1.60±0.39^bc^ | 3.96±0.55^a^ | 2.32±0.43^b^ |
|  | MY1△*hcp* | 0.19±0.04^d^ | 2.34±0.34 | 0.80±0.09 | 5.49±1.72 | 3.17±0.52 | 3.51±0.72 | 3.40±0.68^a^ | 1.70±1.01^b^ | 2.55±0.54^b^ |
| 6 hpi | MY1 | 0.69±0.07^b^ | 7.48±2.16 | 0.26±0.07 | 3.27±0.80 | 1.37±0.36 | 1.41±0.52 | 1.20±0.37^bc^ | 1.36±0.46^b^ | 1.14±0.12^b^ |
|  | MY1△*hcp* | 0.38±0.05^c^ | 3.55±0.85 | 0.40±0.06 | 4.90±1.29 | 1.54±0.40 | 2.60±0.53 | 2.41±0.44^ab^ | 2.09±1.24^ab^ | 1.49±0.78^b^ |
| 9 hpi | MY1 | 0.89±0.01^a^ | 12.28±4.26 | 0.19±0.05 | 6.50±3.06 | 1.29±0.40 | 4.51±1.01 | 3.71±0.62^a^ | 1.47±0.38^b^ | 1.52±0.12^b^ |
|  | MY1△*hcp* | 0.77±0.07^ab^ | 11.25±3.50 | 0.28±0.06 | 5.64±2.51 | 1.25±0.40 | 4.25±0.99 | 3.40±0.68^a^ | 2.25±0.76^ab^ | 4.49±1.21^a^ |
| Time | 0 hpi | 0.02±0.01 | 1.00±0.07^c^ | 1.00±0.07^a^ | 1.01±0.10^b^ | 1.01±0.17^b^ | 1.02±0.20^c^ | 1.02±0.19 | 1.01±0.18 | 1.01±0.16 |
|  | 3 hpi | 0.32±0.16 | 4.43±2.37^b^ | 0.67±0.16^b^ | 3.98±2.00^a^ | 2.81±0.57^a^ | 2.81±0.98^b^ | 2.50±1.10 | 2.83±1.43 | 2.43±0.46 |
|  | 6 hpi | 0.53±0.18 | 5.51±2.61^b^ | 0.33±0.10^c^ | 4.09±1.31^a^ | 1.46±0.35^b^ | 2.00±0.80^b^ | 1.81±0.76 | 1.72±0.93 | 1.31±0.54 |
|  | 9 hpi | 0.83±0.08 | 11.76±3.53^a^ | 0.24±0.07^c^ | 6.07±2.55^a^ | 1.27±0.36^b^ | 4.38±0.91^a^ | 3.55±0.61 | 1.86±0.69 | 3.01±1.80 |
| Treatment | MY1 | 0.51±0.34 | 6.82±4.67^a^ | 0.50±0.34^b^ | 3.31±2.51 | 1.53±0.64 | 2.26±1.53^b^ | 1.88±1.18 | 1.95±1.27 | 1.50±0.57 |
|  | MY1△*hcp* | 0.34±0.30 | 4.53±4.43^b^ | 0.62±0.31^a^ | 4.26±2.43 | 1.74±0.95 | 2.84±1.39^a^ | 2.56±1.12 | 1.76±0.91 | 2.39±1.55 |
|  | *P*-Value（Two-Way ANOVA） | | | | | | | | | |
| Treatment | | 0.000 | 0.019 | 0.001 | 0.171 | 0.177 | 0.049 | 0.004 | 0.526 | 0.002 |
| Time | | 0.000 | 0.000 | 0.000 | 0.001 | 0.000 | 0.000 | 0.000 | 0.003 | 0.000 |
| Treatment * Time | | 0.001 | 0.270 | 0.065 | 0.210 | 0.298 | 0.113 | 0.005 | 0.005 | 0.001 |

Table S1. The apoptosis and immune response between MY1-infected and MY1△*hcp*-infected dGCs.

Note: ^a,b,c,d,e^ Means ± SE with different superscript are significantly different in the same line (*P*<0.05).
