# Supplementary material for: The salmonella effector Hcp modulates infection response, and affects salmonella adhesion and egg contamination incidences in ducks
Source: Front Cell Infect Microbiol. 2022 Oct 3;12:948237. doi: 10.3389/fcimb.2022.948237 (PMC9575552; doi:10.3389/fcimb.2022.948237)
Supplement: Supplementary Table S4 — Quantitative verification of key proteins by parallel reaction monitoring technology (PRM). [file Table_4.docx]

**Table S4 Quantitative verification of key proteins by parallel reaction monitoring technology (PRM)**

| Protein Accession | Protein Gene | Control Relative Abundance (PRM) | Control Relative Abundance (LQ) | MY1 Relative Abundance (PRM) | MY1 Relative Abundance (LQ) | MY1_hcp Relative Abundance (PRM) | MY1_hcp Relative Abundance (LQ) |
| --- | --- | --- | --- | --- | --- | --- | --- |
| Unigene1262_def_Gene.36002 | STAT3 | 1.20±0.54 | 1.13±0.01 | 0.85±0.16 | 0.74±0.01 | 1.09±0.08 | 1.14±0.01 |
| CL1974.Contig2_def_Gene.18159 | BTF3 | 1.40±0.07 | 1.16±0.08 | 0.79±0.20 | 0.74±0.01 | 1.01±0.12 | 1.27±0.09 |
| CL490.Contig4_def_Gene.5516 | CREBBP | 0.91±0.04 | 0.81±0.07 | 1.15±0.17 | 1.28±0.08 | 0.84±0.05 | 0.89±0.01 |
| Unigene36191_def_Gene.56888 | AKT1 | 0.97±0.05 | 1.02±0.03 | 0.72±0.05 | 0.68±0.06 | 1.45±0.09 | 1.28±0.02 |
| CL1534.Contig3_def_Gene.14851 | PKN2 | 1.09±0.13 | 1.09±0.03 | 0.91±0.22 | 0.62±0.09 | 1.03±0.14 | 1.38±0.06 |
| CL4569.Contig2_def_Gene.32339 | DOCK1 | 1.24±0.40 | 1.18±0.02 | 0.70±0.06 | 0.60±0.02 | 0.44±0.36 | 1.16±0.04 |
| CL4543.Contig2_def_Gene.32201 | NEK7 | 0.96±0.05 | 1.04±0.04 | 0.95±0.21 | 0.65±0.07 | 1.20±0.12 | 1.27±0.01 |
| Unigene6849_def_Gene.39389 | CASP7 | 1.06±0.07 | 1.17±0.08 | 0.87±0.22 | 0.59±0.09 | 1.15±0.05 | 1.21±0.13 |
| CL3170.Contig1_def_Gene.25521 | MAPK9 | 1.05±0.16 | 1.05±0.08 | 0.78±0.10 | 0.69±0.05 | 1.25±0.21 | 1.37±0.12 |
| CL3292.Contig1_def_Gene.26167 | TP53I3 | 0.98±0.13 | 0.83±0.09 | 0.97±0.16 | 0.64±0.01 | 1.56±0.26 | 1.39±0.02 |
| CL3320.Contig1_def_Gene.26359 | MAPK14 | 0.96±0.23 | 1.25±0.03 | 0.87±0.16 | 0.59±0.01 | 1.19±0.11 | 1.11±0.04 |
